# Reticulated acanthoma with sebaceous differentiation: A case report

**DOI:** 10.1097/MD.0000000000046426

**Published:** 2025-12-12

**Authors:** Wan-Ting Yu, Qian Zhang, Hong Shen, Ze-Hu Liu

**Affiliations:** a Department of Dermatology, Hangzhou Third People’s Hospital, Hangzhou Third Hospital Affiliated to Zhejiang Chinese Medical University, Hangzhou, Zhejiang Province, China.

**Keywords:** DNA mismatch repair proteins, immunohistochemistry, Muir-Torre syndrome, reticulated acanthoma with sebaceous differentiation, sebaceous gland neoplasms

## Abstract

**Rationale:**

Reticulated acanthoma with sebaceous differentiation (RASD) is a rare and benign cutaneous neoplasm that is characterized by reticulated epidermal hyperplasia and differentiation of mature sebaceous cells. Due to the scarcity of this condition, the potential association with Muir-Torre syndrome (MTS) remains the subject of debate.

**Patient concerns:**

A 52-year-old Chinese woman presented with a 3-year history of a solitary papule on the right buttock.

**Diagnoses:**

Histopathology provided a confirmed diagnosis of RASD. Immunohistochemical analysis demonstrated uniform nuclear positivity for DNA mismatch repair proteins (*MLH1, MSH2, MSH6*, and PMS2). The absence of visceral malignancies and a negative family history, combined with the immunohistochemical results, provided no evidence for an association with MTS.

**Interventions:**

The lesion was completely excised with negative margins.

**Outcomes:**

No tumor recurrence was observed during 20 months of follow-up.

**Lessons:**

This rare case highlights the critical role of histopathological examination. Given the limited body of documented evidence for an association between RASD and MTS, documented, albeit limited, baseline immunohistochemistry and colonoscopy should always be considered to rule out MTS in patients diagnosed with RASD.

## 1. Introduction

Reticulated acanthoma with sebaceous differentiation (RASD) is a rare and benign epidermal tumor that is characterized by reticulated epidermal hyperplasia and sebocytic maturation, first described by Steffen and Ackerman.^[1]^ While RASD has been sporadically reported in association with Muir-Torre syndrome (MTS), this relationship remains contentious. MTS, is an autosomal dominant form of genodermatosis and manifests as multiple sebaceous neoplasms and a strong association with visceral malignancies, particularly colorectal carcinoma. The pathogenesis of MTS is linked to germline mutations in DNA *MMR* genes, particularly *MLH1, MSH2, MSH6*, and *PMS2*.^[2]^ Here, we present a new case of RASD, analyze the potential link to MTS, and emphasize the importance of systemic screening in clinical practice.

## 2. Case description

**Figure 1.**
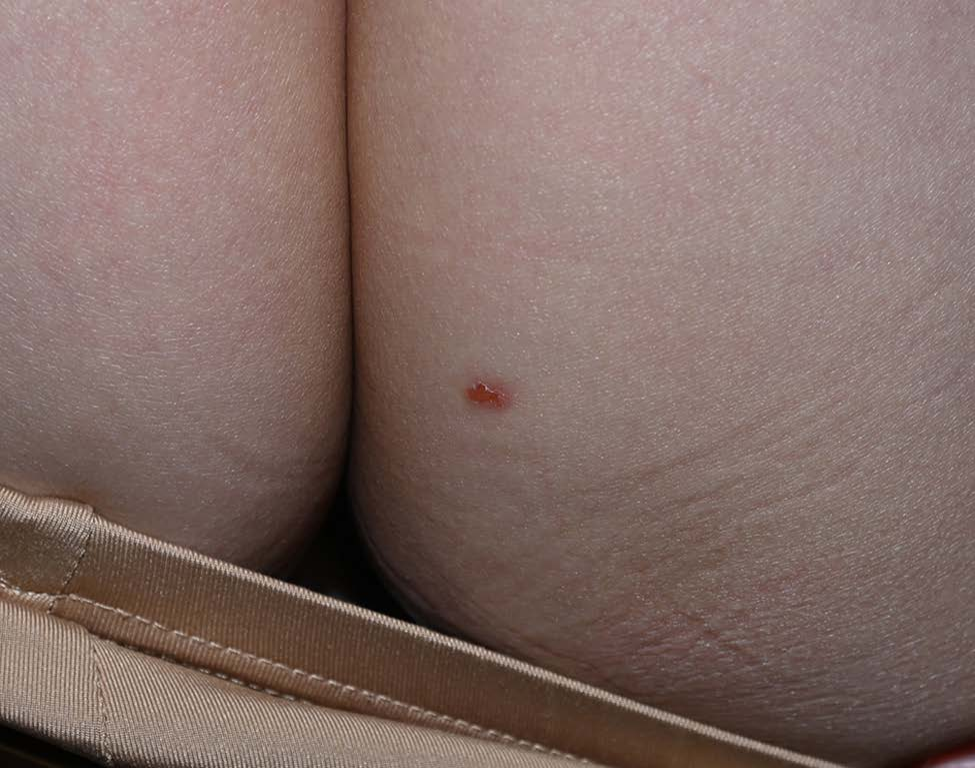
A 6- mm erythematous papule on the right buttock of a 52-yr-old female.

**Figure 2.**
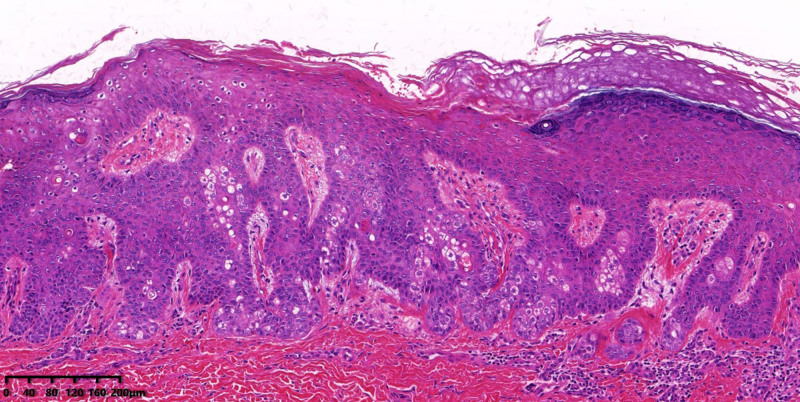
Histopathological examination revealed hyperkeratosis, parakeratosis, acanthosis with anastomosing rete ridges forming a reticulated pattern, and clusters of mature sebocytes in the mid-to-lower epidermis (H&E staining, ×200).

**Figure 3.**
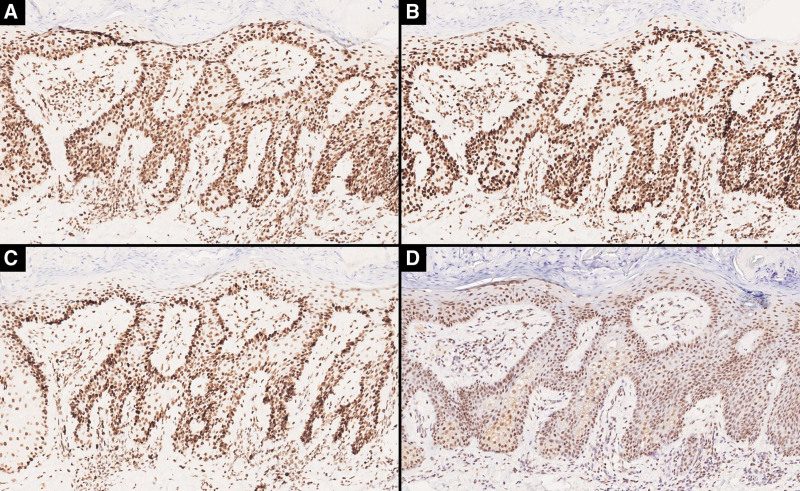
Immunohistochemistry showed the preserved nuclear expression of (a) MLH1, (b) MSH2, (c) MSH6, and (d) PMS2 (×200).

A 52-year-old Chinese woman presented with a 3-year history of a solitary, mildly pruritic and tender, 6-mm erythematous papule on the right buttock (Fig. 1). Physical examination revealed no systemic abnormalities. Histopathological findings of the excised lesion demonstrated hyperkeratosis, parakeratosis, acanthosis with anastomosing rete ridges forming a reticulated pattern, and clusters of mature sebocytes in the mid-to-lower epidermis (Fig. 2). Immunohistochemistry revealed preserved nuclear expression of MLH1, MSH2, MSH6 and PMS2 (Fig. 3). A detailed systemic evaluation detected no visceral malignancies and the patient reported no family history of malignancy. At the 20-month follow-up, no evidence of recurrence was observed. The patient continues to be followed up. This case study was approved by the Institutional Review Board of Hangzhou Third People’s Hospital.

## 3. Discussion

Initially described by various diagnostic terms such as “superficial epithelioma with sebaceous differentiation.” RASD was redefined by Steffen and Ackerman to emphasize its histopathological hallmarks of reticulated epidermal hyperplasia and sebocytic maturation.^[3,4]^ The precise etiology of RASD remains unknown. Lesions typically present as yellowish-red papules, plaques, or nodules on the trunk, head, or neck and are occasionally bordered by peripheral hyperpigmentation. While most patients are asymptomatic, mild pruritus or tenderness may occur. The clinical course typically spans decades. Clinically, it is important that RASD must be differentiated from eczema, Bowen disease, basal cell carcinoma, and melanoma. Histopathological examination reveals key diagnostic features, including epidermal acanthosis with anastomosing rete ridges that form a reticulated architecture and clusters of mature sebocytes in the mid-to-lower epidermis. Differential histological diagnoses include sebaceoma, sebaceous nevus, seborrheic keratosis and poroma.^[1,5]^ Dermoscopy may facilitate diagnosis by revealing yellowish reticular patterns.^[6,7]^ However, melanoma-mimicking features in RASD have also been reported.^[8]^

The precise relationship between RASD and MTS, an autosomal dominant genodermatosis associated with DNA mismatch repair gene defects, remains contentious. Immunohistochemical analysis of DNA *MMR* is critical for evaluating potential association with MTS. The preservation of nuclear expression disputes MTS comorbidity, whereas the loss of any marker raises suspicion. In the present case, tumor cells exhibited intact expression of all 4 markers (*MLH1*, *MSH2*, *MSH6* and *PMS2*), and there was no personal or family history of visceral malignancy. Although most cases of RASD lack an association with MTS, rare exceptions justify systematic screening.^[9]^ Table 1 summarizes the difference between published RASD cases associated with MTS and those that were not. Baseline evaluation with immunohistochemistry for DNA *MMR* proteins is recommended, followed by colonoscopy if *MMR* deficiency is detected.

**Table 1:** Clinical differences in reticulated acanthoma with sebaceous differentiation with or without Muir-Torre syndrome.

Feature	RASD Without MTS	RASD With MTS
Prevalence	~90% of cases	~10% (rare)
MMR IHC	Intact (MSH2, MSH6, MLH1, PMS2)	Loss (e.g., MSH6)
Family history	Negative	Positive (MTS)
Multiple sebaceous tumors	Absent	Present (e.g., sebaceous adenoma)
Visceral malignancy risk	Low	High (colorectal, endometrial)
Management	Excision + routine follow-up	Genetic testing, cancer screening

IHC = immunohistochemistry, MMR = mismatch repair proteins, MTS = Muir-Torre syndrome, RASD = reticulated acanthoma with sebaceous differentiation.

Surgical excision remains the cornerstone of RASD management and can effectively by preventing malignant transformation. Although RASD is benign, a documented case of Bowen disease arising within a RASD lesion highlights the importance of complete resection.^[7,10]^

## 4. Conclusions

RASD is a histopathologically distinct entity that requires careful differentiation from both benign and malignant sebaceous tumors. Surgical intervention is the primary form of treatment for RASD, and the prognosis is generally favorable. The association of some RASD cases with MTS highlights the importance of screening for DNA *MMR* gene mutations and visceral malignancies in clinical practice. Long-term follow-up is essential for patients with concurrent MTS.

## Acknowledgments

The authors would like to express their gratitude to EditSprings (https://www.editsprings.com) for the expert linguistic services provided.

## Author contributions

**Conceptualization:** Ze-Hu Liu.

**Data curation:** Wan-Ting Yu.

**Formal analysis:** Ze-Hu Liu.

**Investigation:** Wan-Ting Yu.

**Methodology:** Ze-Hu Liu.

**Supervision:** Hong Shen, Ze-Hu Liu.

**Validation:** Qian Zhang, Hong Shen.

**Visualization:** Qian Zhang.

**Writing – original draft:** Wan-Ting Yu.

**Writing – review & editing:** Hong Shen, Ze-Hu Liu.
